# 2-[(5′-Chloro-1,1′:3′,1′′-terphenyl-4′-yl)imino]­acenaphthylen-1(2*H*)-one

**DOI:** 10.1107/S1600536813008015

**Published:** 2013-04-05

**Authors:** Zhengyin Du, Fushou Che, Yufei Yan, Wei Liu

**Affiliations:** a Key Laboratory of Eco-Environment-Related Polymer Materials of the Ministry of Education, Key Laboratory of Polymer Materials of Gansu Province, College of Chemistry & Chemical Engineering, Northwest Normal University, Lanzhou 730070, People’s Republic of China

## Abstract

The title compound, C_30_H_18_ClNO, is a product of the condensation reaction of acenaphthyl­ene-1,2-dione and 5′-chloro-1,1′:3′,1′′-terphenyl-4′-amine. The acenaphthyl­ene fragment and two terminal phenyl rings are rotated relative to the central benzene ring by 72.2 (3), 43.2 (3) and 41.2 (3)°, respectively. This mol­ecular conformation is supported by weak C—H⋯π inter­actions. In the crystal, mol­ecules form centrosymmetric dimers by the stacking inter­actions between two neighboring acenaphthyl­ene fragments, with an inter­planar distance of 3.365 (3) Å. The dimers are bound to each other by weak C—H⋯N and C—H⋯π inter­actions, forming a three-dimensional framework.

## Related literature

For background to applications of Schiff bases, see: Lozier *et al.* (1975 ▶); Kargar *et al.* (2009 ▶); Yeap *et al.* (2009 ▶). For related structures, see: Higuchi *et al.* (2001 ▶); Manseong *et al.* (2006 ▶); Vitor *et al.* (2008 ▶).
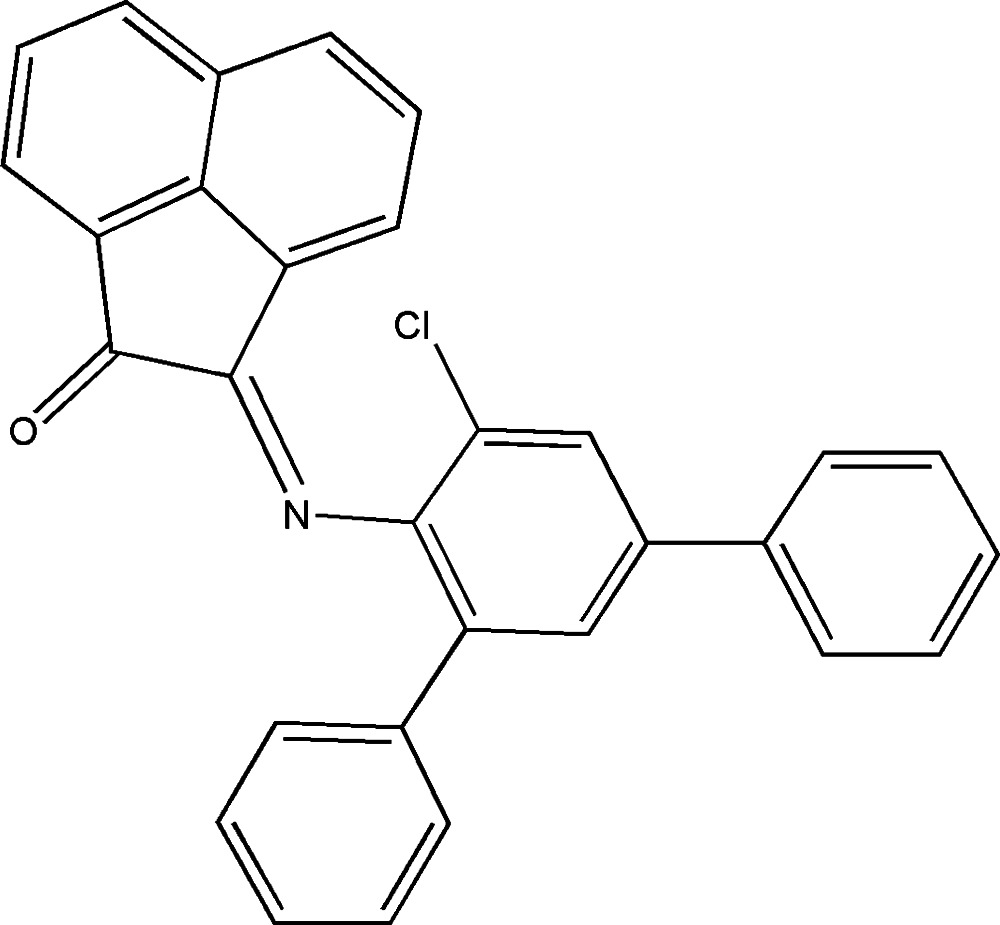


## Experimental

### 

#### Crystal data

C_30_H_18_ClNO
*M* *_r_* = 443.90
Monoclinic, 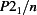

*a* = 12.4929 (6) Å
*b* = 10.8699 (7) Å
*c* = 16.0758 (8) Å
β = 91.864 (5)°
*V* = 2181.9 (2) Å^3^
*Z* = 4
Mo *K*α radiation
μ = 0.20 mm^−1^
*T* = 100 K
0.32 × 0.28 × 0.25 mm

#### Data collection

Agilent SuperNova (Dual, Cu at zero, Eos) diffractometer
Absorption correction: multi-scan (*CrysAlis PRO*; Agilent, 2012 ▶) *T*_min_ = 0.866, *T*_max_ = 1.000
8827 measured reflections
4461 independent reflections
3151 reflections with *I* > 2σ(*I*)
*R*_int_ = 0.041

#### Refinement

*R*[*F* ^2^ > 2σ(*F* ^2^)] = 0.058
*wR*(*F* ^2^) = 0.121
*S* = 1.09
4461 reflections
298 parameters
H-atom parameters constrained
Δρ_max_ = 0.50 e Å^−3^
Δρ_min_ = −0.28 e Å^−3^

### 

Data collection: *CrysAlis PRO* (Agilent, 2012 ▶); cell refinement: *CrysAlis PRO*; data reduction: *CrysAlis PRO*; program(s) used to solve structure: *SHELXTL* (Sheldrick, 2008 ▶); program(s) used to refine structure: *SHELXTL*; molecular graphics: *OLEX2* (Dolomanov *et al.*, 2009 ▶); software used to prepare material for publication: *OLEX2*.

## Supplementary Material

Crystal structure: contains datablock(s) global, I. DOI: 10.1107/S1600536813008015/kq2002sup1.cif

Structure factors: contains datablock(s) I. DOI: 10.1107/S1600536813008015/kq2002Isup2.hkl

Supplementary material file. DOI: 10.1107/S1600536813008015/kq2002Isup3.cml

Additional supplementary materials:  crystallographic information; 3D view; checkCIF report

## Figures and Tables

**Table 1:** Hydrogen-bond geometry (Å, °) *Cg*1 and *Cg*2 are the centroids of the C25–C30 and C19–C24 rings, respectively.

*D*—H⋯*A*	*D*—H	H⋯*A*	*D*⋯*A*	*D*—H⋯*A*
C24—H24⋯N2^i^	0.93	2.59	3.338 (3)	138
C4—H4⋯*Cg*1^ii^	0.93	2.74	3.551 (3)	147
C6—H6⋯*Cg*2^iii^	0.93	2.92	3.647 (3)	136

Symmetry codes: (i) 
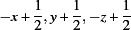
; (ii) 
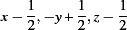
; (iii) 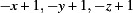.

**Fig. 1.**
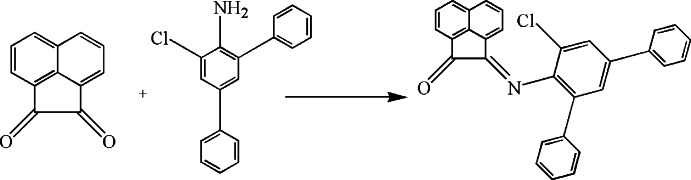
A condensation reaction of acenaphthylene-1,2-dione and 5'-chloro-1,1':3',1''-terphenyl-4'-amine.

**Fig. 2.**
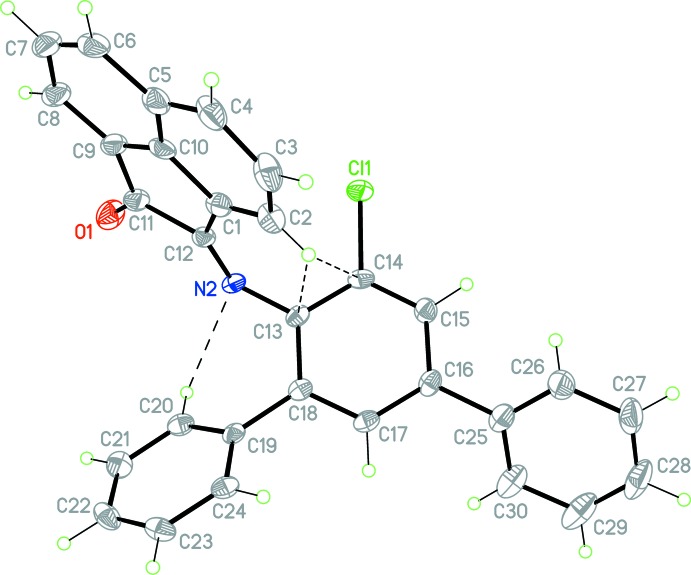
Molecular structure of I. Displacement ellipsoids are shown at the 40% probability level. H atoms are presented as small spheres of arbitrary radius. The dashed lines indicate the intramolecular C—H···N and C—H···π(C=C) hydrogen bonds.

## supplementary crystallographic information

### Comment

Schiff bases often exhibit various biological activities, for example, anti-inflammatory, antibacterial, anticancer and antitoxic properties (Lozier *et al.*, 1975). They have also been used as versatile ligands in coordination chemistry (Kargar *et al.*, 2009; Yeap *et al.*, 2009). The present work describes the structure of a new Schiff base, C_30_H_18_ClNO, (I) obtained by the condensation reaction of acenaphthylene-1,2-dione and 5'-chloro-1,1':3',1''-terphenyl-4'-amine (Figure 1).

The geometrical parameters of I are in good accordance with those found in the related compounds (Higuchi *et al.*, 2001; Manseong *et al.*, 2006; Vitor *et al.*, 2008). The acenaphthylene fragment and two terminal phenyl rings in I are rotated in relative to the central benzene ring by 72.2 (3), 43.2 (3) and 41.2 (3)°, respectively (Figure 2). The molecular conformation of I is supported by the weak intramolecular C20—H20···N2 (Table 1) and C2—H2···π(C13=C14) [H2···C13 2.76 Å, H2···C14 2.80 Å] hydrogen bonding interactions (Figure 2).

In the crystal, the molecules form centrosymmetric dimers by the stacking interactions between two neighboring acenaphthylene fragments, with the interplane distance of 3.365 (3) Å. The dimers are bound to each other by the weak C24—H24···N2^i^, C4—H4···Cg1^ii^ (Cg1 is the centroid of the C25/C26/C27/C28/C29/C30 ring) and C6—H6···Cg2^iii^ (Cg2 is the centroid of the C19/C20/C21/C22/C23/C24 ring) hydrogen bonding interactions (Table 1) into 3-dimensional framework. Symmetry codes: (i) –*x*+1/2, *y*+1/2, –*z*+1/2; (ii) *x*–1/2, –*y*+1/2, *z*–1/2; (iii) –*x*+1, –*y*+1, –*z*+1.

### Experimental

Formic acid (1 mL) was added to a stirred solution of acenaphthenequinone (1.2 mmol) and 2-chloro-4,6-diphenylaniline (1.2 mmol) in methanol (20 mL). The mixture was refluxed for 24 h, then cooled, and the precipitate was separated by filtration. The solid was recrystallized from dichloromethane/cyclohexane (*v*/*v* = 8:1), washed with cold ethanol and dried under vacuum to give the title compound I. Yield is 0.15 g (83%). Crystals of I suitable for X-ray structure determination were grown from a cyclohexane/dichloromethane (1:2, *v*/*v*) solution. Anal. Calcd. for C_30_H_18_ClNO: C, 81.17; H, 4.09; N,3.16. Found: C, 81.11; H, 4.19; N, 3.12.

### Refinement

All hydrogen atoms were placed in the calculated positions with C—H = 0.93 Å and refined in the riding model with fixed isotropic displacement parameters [*U*_iso_(H) = 1.2*U*_eq_(C)].

### Crystal data

**Table:**

C_30_H_18_ClNO	*F*(000) = 920
*M**_r_* = 443.90	*D*_x_ = 1.351 Mg m^−^^3^
Monoclinic, *P*2_1_/*n*	Mo *K*α radiation, λ = 0.7107 Å
*a* = 12.4929 (6) Å	Cell parameters from 2796 reflections
*b* = 10.8699 (7) Å	θ = 3.1–28.4°
*c* = 16.0758 (8) Å	µ = 0.20 mm^−^^1^
β = 91.864 (5)°	*T* = 100 K
*V* = 2181.9 (2) Å^3^	Block, clear light-yellow
*Z* = 4	0.32 × 0.28 × 0.25 mm

### Data collection

**Table:**

Agilent SuperNova (Dual, Cu at zero, Eos) diffractometer	4461 independent reflections
Radiation source: SuperNova (Mo) X-ray Source	3151 reflections with *I* > 2σ(*I*)
Mirror monochromator	*R*_int_ = 0.041
Detector resolution: 16.0733 pixels mm^-1^	θ_max_ = 26.4°, θ_min_ = 3.2°
ω scans	*h* = −15→15
Absorption correction: multi-scan (*CrysAlis PRO*; Agilent, 2012)	*k* = −13→11
*T*_min_ = 0.866, *T*_max_ = 1.000	*l* = −19→20
8827 measured reflections	

### Refinement

**Table:**

Refinement on *F*^2^	Primary atom site location: structure-invariant direct methods
Least-squares matrix: full	Secondary atom site location: difference Fourier map
*R*[*F*^2^ > 2σ(*F*^2^)] = 0.058	Hydrogen site location: inferred from neighbouring sites
*wR*(*F*^2^) = 0.121	H-atom parameters constrained
*S* = 1.09	*w* = 1/[σ^2^(*F*_o_^2^) + (0.031*P*)^2^ + 0.7817*P*] where *P* = (*F*_o_^2^ + 2*F*_c_^2^)/3
4461 reflections	(Δ/σ)_max_ < 0.001
298 parameters	Δρ_max_ = 0.50 e Å^−^^3^
0 restraints	Δρ_min_ = −0.28 e Å^−^^3^

### Special details

**Table:**

Geometry. All e.s.d.'s (except the e.s.d. in the dihedral angle between two l.s. planes) are estimated using the full covariance matrix. The cell e.s.d.'s are taken into account individually in the estimation of e.s.d.'s in distances, angles and torsion angles; correlations between e.s.d.'s in cell parameters are only used when they are defined by crystal symmetry. An approximate (isotropic) treatment of cell e.s.d.'s is used for estimating e.s.d.'s involving l.s. planes.
Refinement. Refinement of *F*^2^ against ALL reflections. The weighted *R*-factor *wR* and goodness of fit *S* are based on *F*^2^, conventional *R*-factors *R* are based on *F*, with *F* set to zero for negative *F*^2^. The threshold expression of *F*^2^ > σ(*F*^2^) is used only for calculating *R*-factors(gt) *etc*. and is not relevant to the choice of reflections for refinement. *R*-factors based on *F*^2^ are statistically about twice as large as those based on *F*, and *R*- factors based on ALL data will be even larger.

### Fractional atomic coordinates and isotropic or equivalent isotropic displacement parameters (Å^2^)

**Table:**

	*x*	*y*	*z*	*U*_iso_*/*U*_eq_	
Cl1	0.04696 (5)	0.31870 (6)	0.48276 (4)	0.02604 (18)	
O1	0.41337 (15)	0.16285 (19)	0.45458 (12)	0.0333 (5)	
N2	0.23922 (15)	0.29536 (19)	0.37546 (11)	0.0179 (5)	
C1	0.29301 (19)	0.4619 (3)	0.47857 (15)	0.0226 (6)	
C2	0.2362 (2)	0.5671 (3)	0.46784 (16)	0.0255 (6)	
H2	0.1824	0.5737	0.4267	0.031*	
C3	0.2617 (2)	0.6671 (3)	0.52178 (18)	0.0335 (7)	
H3	0.2249	0.7408	0.5143	0.040*	
C4	0.3393 (2)	0.6593 (3)	0.58509 (17)	0.0334 (7)	
H4	0.3515	0.7262	0.6201	0.040*	
C5	0.3989 (2)	0.5525 (3)	0.59681 (16)	0.0276 (7)	
C6	0.4832 (2)	0.5294 (3)	0.65685 (16)	0.0330 (7)	
H6	0.5019	0.5901	0.6954	0.040*	
C7	0.5368 (2)	0.4199 (3)	0.65874 (16)	0.0352 (8)	
H7	0.5911	0.4087	0.6990	0.042*	
C8	0.5140 (2)	0.3233 (3)	0.60276 (15)	0.0317 (7)	
H8	0.5527	0.2502	0.6045	0.038*	
C9	0.4309 (2)	0.3422 (3)	0.54485 (15)	0.0249 (6)	
C10	0.3752 (2)	0.4534 (3)	0.54261 (15)	0.0250 (6)	
C11	0.3865 (2)	0.2656 (3)	0.47682 (15)	0.0247 (6)	
C12	0.29469 (18)	0.3418 (2)	0.43510 (14)	0.0186 (6)	
C13	0.15077 (18)	0.3582 (2)	0.33866 (14)	0.0176 (5)	
C14	0.05499 (19)	0.3719 (2)	0.38076 (14)	0.0195 (6)	
C15	−0.03509 (19)	0.4241 (2)	0.34434 (15)	0.0213 (6)	
H15	−0.0974	0.4306	0.3741	0.026*	
C16	−0.03305 (19)	0.4674 (2)	0.26273 (15)	0.0208 (6)	
C17	0.06207 (18)	0.4559 (2)	0.22035 (15)	0.0195 (6)	
H17	0.0645	0.4862	0.1663	0.023*	
C18	0.15357 (18)	0.4010 (2)	0.25532 (14)	0.0169 (5)	
C19	0.25352 (18)	0.3931 (2)	0.20691 (14)	0.0176 (5)	
C20	0.31803 (19)	0.2884 (2)	0.20722 (14)	0.0211 (6)	
H20	0.2982	0.2193	0.2372	0.025*	
C21	0.4119 (2)	0.2865 (3)	0.16287 (15)	0.0250 (6)	
H21	0.4542	0.2160	0.1632	0.030*	
C22	0.4425 (2)	0.3883 (3)	0.11853 (15)	0.0265 (6)	
H22	0.5062	0.3874	0.0902	0.032*	
C23	0.3783 (2)	0.4918 (3)	0.11635 (15)	0.0247 (6)	
H23	0.3984	0.5603	0.0859	0.030*	
C24	0.28386 (19)	0.4937 (2)	0.15946 (14)	0.0209 (6)	
H24	0.2403	0.5631	0.1566	0.025*	
C25	−0.1326 (2)	0.5199 (3)	0.22308 (17)	0.0258 (6)	
C26	−0.2001 (2)	0.5956 (3)	0.26780 (18)	0.0335 (7)	
H26	−0.1807	0.6176	0.3221	0.040*	
C27	−0.2959 (2)	0.6385 (3)	0.2324 (2)	0.0415 (8)	
H27	−0.3399	0.6897	0.2625	0.050*	
C28	−0.3251 (2)	0.6047 (3)	0.1522 (2)	0.0447 (9)	
H28	−0.3900	0.6316	0.1287	0.054*	
C29	−0.2592 (2)	0.5316 (3)	0.1070 (2)	0.0434 (9)	
H29	−0.2794	0.5096	0.0529	0.052*	
C30	−0.1619 (2)	0.4899 (3)	0.14165 (18)	0.0338 (7)	
H30	−0.1167	0.4420	0.1101	0.041*	

### Atomic displacement parameters (Å^2^)

**Table:**

	*U*^11^	*U*^22^	*U*^33^	*U*^12^	*U*^13^	*U*^23^
Cl1	0.0262 (3)	0.0333 (4)	0.0187 (3)	−0.0013 (3)	0.0026 (2)	0.0074 (3)
O1	0.0344 (11)	0.0298 (12)	0.0354 (11)	0.0085 (10)	−0.0043 (8)	0.0040 (9)
N2	0.0197 (10)	0.0197 (12)	0.0139 (10)	−0.0025 (10)	−0.0030 (8)	0.0026 (9)
C1	0.0201 (12)	0.0284 (16)	0.0193 (13)	−0.0087 (13)	0.0024 (10)	−0.0033 (11)
C2	0.0239 (13)	0.0242 (16)	0.0285 (14)	−0.0040 (13)	0.0047 (11)	−0.0062 (12)
C3	0.0247 (14)	0.0299 (18)	0.0461 (18)	−0.0004 (14)	0.0070 (12)	−0.0112 (14)
C4	0.0246 (14)	0.041 (2)	0.0352 (16)	−0.0075 (15)	0.0095 (12)	−0.0183 (14)
C5	0.0208 (13)	0.0387 (19)	0.0235 (14)	−0.0099 (14)	0.0055 (11)	−0.0036 (13)
C6	0.0295 (15)	0.050 (2)	0.0195 (14)	−0.0194 (16)	0.0031 (11)	−0.0103 (14)
C7	0.0259 (15)	0.058 (2)	0.0209 (14)	−0.0123 (16)	−0.0051 (11)	0.0032 (14)
C8	0.0264 (14)	0.048 (2)	0.0201 (13)	−0.0138 (15)	−0.0022 (10)	0.0100 (13)
C9	0.0231 (13)	0.0350 (18)	0.0165 (12)	−0.0062 (14)	−0.0005 (10)	0.0046 (12)
C10	0.0212 (13)	0.0396 (18)	0.0145 (12)	−0.0137 (14)	0.0059 (10)	−0.0023 (12)
C11	0.0251 (14)	0.0293 (17)	0.0197 (13)	−0.0020 (14)	0.0009 (10)	0.0075 (12)
C12	0.0188 (12)	0.0219 (15)	0.0150 (12)	−0.0035 (12)	0.0013 (9)	0.0020 (11)
C13	0.0212 (12)	0.0141 (13)	0.0170 (12)	−0.0008 (11)	−0.0048 (9)	−0.0020 (10)
C14	0.0250 (13)	0.0184 (15)	0.0150 (12)	−0.0048 (12)	−0.0005 (10)	0.0031 (10)
C15	0.0174 (12)	0.0217 (15)	0.0248 (13)	−0.0032 (12)	0.0014 (10)	0.0031 (11)
C16	0.0165 (12)	0.0186 (15)	0.0268 (14)	−0.0040 (12)	−0.0043 (10)	0.0020 (11)
C17	0.0235 (13)	0.0180 (14)	0.0167 (12)	−0.0036 (12)	−0.0039 (10)	0.0014 (11)
C18	0.0201 (12)	0.0135 (13)	0.0168 (12)	−0.0033 (11)	−0.0028 (9)	−0.0026 (10)
C19	0.0186 (12)	0.0204 (14)	0.0134 (11)	−0.0007 (12)	−0.0050 (9)	−0.0044 (10)
C20	0.0266 (13)	0.0191 (15)	0.0174 (12)	−0.0025 (12)	−0.0036 (10)	−0.0020 (11)
C21	0.0273 (14)	0.0236 (16)	0.0239 (14)	0.0030 (13)	−0.0003 (11)	−0.0086 (12)
C22	0.0242 (14)	0.0339 (18)	0.0218 (14)	−0.0070 (14)	0.0071 (11)	−0.0105 (12)
C23	0.0313 (14)	0.0247 (16)	0.0183 (13)	−0.0079 (14)	0.0039 (10)	−0.0023 (11)
C24	0.0269 (13)	0.0205 (15)	0.0151 (12)	−0.0011 (12)	−0.0037 (10)	−0.0019 (11)
C25	0.0197 (13)	0.0257 (16)	0.0318 (15)	−0.0054 (13)	−0.0030 (11)	0.0104 (12)
C26	0.0249 (14)	0.0405 (19)	0.0354 (16)	0.0011 (15)	0.0070 (12)	0.0145 (14)
C27	0.0211 (14)	0.047 (2)	0.058 (2)	0.0073 (15)	0.0114 (14)	0.0223 (17)
C28	0.0195 (14)	0.045 (2)	0.069 (2)	−0.0037 (16)	−0.0114 (15)	0.0275 (19)
C29	0.0426 (18)	0.033 (2)	0.052 (2)	−0.0058 (17)	−0.0250 (15)	0.0092 (16)
C30	0.0345 (16)	0.0253 (17)	0.0405 (17)	−0.0008 (15)	−0.0146 (13)	0.0026 (14)

### Geometric parameters (Å, º)

**Table:**

Cl1—C14	1.745 (2)	C15—C16	1.395 (3)
O1—C11	1.223 (3)	C16—C17	1.394 (3)
N2—C12	1.269 (3)	C16—C25	1.492 (3)
N2—C13	1.412 (3)	C17—H17	0.9300
C1—C2	1.354 (4)	C17—C18	1.392 (3)
C1—C10	1.433 (3)	C18—C19	1.495 (3)
C1—C12	1.481 (4)	C19—C20	1.395 (3)
C2—H2	0.9300	C19—C24	1.393 (3)
C2—C3	1.420 (4)	C20—H20	0.9300
C3—H3	0.9300	C20—C21	1.392 (3)
C3—C4	1.385 (4)	C21—H21	0.9300
C4—H4	0.9300	C21—C22	1.378 (4)
C4—C5	1.389 (4)	C22—H22	0.9300
C5—C6	1.427 (4)	C22—C23	1.381 (4)
C5—C10	1.411 (4)	C23—H23	0.9300
C6—H6	0.9300	C23—C24	1.388 (3)
C6—C7	1.366 (4)	C24—H24	0.9300
C7—H7	0.9300	C25—C26	1.394 (4)
C7—C8	1.407 (4)	C25—C30	1.387 (4)
C8—H8	0.9300	C26—H26	0.9300
C8—C9	1.387 (3)	C26—C27	1.389 (4)
C9—C10	1.394 (4)	C27—H27	0.9300
C9—C11	1.469 (4)	C27—C28	1.378 (5)
C11—C12	1.550 (4)	C28—H28	0.9300
C13—C14	1.402 (3)	C28—C29	1.370 (5)
C13—C18	1.420 (3)	C29—H29	0.9300
C14—C15	1.374 (3)	C29—C30	1.396 (4)
C15—H15	0.9300	C30—H30	0.9300
			
C12—N2—C13	121.6 (2)	C15—C16—C25	119.4 (2)
C2—C1—C10	120.5 (2)	C17—C16—C15	118.1 (2)
C2—C1—C12	134.4 (2)	C17—C16—C25	122.5 (2)
C10—C1—C12	105.1 (2)	C16—C17—H17	118.6
C1—C2—H2	121.2	C18—C17—C16	122.9 (2)
C1—C2—C3	117.5 (2)	C18—C17—H17	118.6
C3—C2—H2	121.2	C13—C18—C19	121.3 (2)
C2—C3—H3	118.7	C17—C18—C13	118.4 (2)
C4—C3—C2	122.7 (3)	C17—C18—C19	120.2 (2)
C4—C3—H3	118.7	C20—C19—C18	122.5 (2)
C3—C4—H4	119.6	C24—C19—C18	119.1 (2)
C3—C4—C5	120.7 (3)	C24—C19—C20	118.4 (2)
C5—C4—H4	119.6	C19—C20—H20	119.8
C4—C5—C6	128.3 (3)	C21—C20—C19	120.4 (2)
C4—C5—C10	117.0 (2)	C21—C20—H20	119.8
C10—C5—C6	114.8 (3)	C20—C21—H21	119.8
C5—C6—H6	119.4	C22—C21—C20	120.4 (3)
C7—C6—C5	121.3 (3)	C22—C21—H21	119.8
C7—C6—H6	119.4	C21—C22—H22	120.1
C6—C7—H7	118.4	C21—C22—C23	119.8 (2)
C6—C7—C8	123.2 (3)	C23—C22—H22	120.1
C8—C7—H7	118.4	C22—C23—H23	119.9
C7—C8—H8	121.6	C22—C23—C24	120.2 (3)
C9—C8—C7	116.8 (3)	C24—C23—H23	119.9
C9—C8—H8	121.6	C19—C24—H24	119.6
C8—C9—C10	120.5 (3)	C23—C24—C19	120.8 (3)
C8—C9—C11	132.2 (3)	C23—C24—H24	119.6
C10—C9—C11	107.2 (2)	C26—C25—C16	121.0 (2)
C5—C10—C1	121.7 (3)	C30—C25—C16	120.3 (3)
C9—C10—C1	114.8 (2)	C30—C25—C26	118.7 (3)
C9—C10—C5	123.5 (2)	C25—C26—H26	119.5
O1—C11—C9	129.4 (3)	C27—C26—C25	120.9 (3)
O1—C11—C12	124.6 (2)	C27—C26—H26	119.5
C9—C11—C12	106.0 (2)	C26—C27—H27	120.2
N2—C12—C1	133.7 (2)	C28—C27—C26	119.5 (3)
N2—C12—C11	119.5 (2)	C28—C27—H27	120.2
C1—C12—C11	106.8 (2)	C27—C28—H28	119.8
N2—C13—C18	120.7 (2)	C29—C28—C27	120.3 (3)
C14—C13—N2	121.2 (2)	C29—C28—H28	119.8
C14—C13—C18	117.9 (2)	C28—C29—H29	119.8
C13—C14—Cl1	119.65 (18)	C28—C29—C30	120.5 (3)
C15—C14—Cl1	117.78 (19)	C30—C29—H29	119.8
C15—C14—C13	122.6 (2)	C25—C30—C29	120.0 (3)
C14—C15—H15	120.0	C25—C30—H30	120.0
C14—C15—C16	120.0 (2)	C29—C30—H30	120.0
C16—C15—H15	120.0		
			
Cl1—C14—C15—C16	−179.6 (2)	C12—C1—C2—C3	−177.2 (3)
O1—C11—C12—N2	2.8 (4)	C12—C1—C10—C5	178.9 (2)
O1—C11—C12—C1	−177.2 (2)	C12—C1—C10—C9	0.9 (3)
N2—C13—C14—Cl1	3.9 (3)	C13—N2—C12—C1	−3.7 (4)
N2—C13—C14—C15	−174.9 (2)	C13—N2—C12—C11	176.2 (2)
N2—C13—C18—C17	176.2 (2)	C13—C14—C15—C16	−0.8 (4)
N2—C13—C18—C19	−6.6 (4)	C13—C18—C19—C20	44.1 (3)
C1—C2—C3—C4	−1.8 (4)	C13—C18—C19—C24	−136.0 (2)
C2—C1—C10—C5	0.7 (4)	C14—C13—C18—C17	1.0 (4)
C2—C1—C10—C9	−177.3 (2)	C14—C13—C18—C19	178.2 (2)
C2—C1—C12—N2	−4.2 (5)	C14—C15—C16—C17	0.0 (4)
C2—C1—C12—C11	175.8 (3)	C14—C15—C16—C25	177.9 (2)
C2—C3—C4—C5	2.3 (4)	C15—C16—C17—C18	1.4 (4)
C3—C4—C5—C6	178.2 (3)	C15—C16—C25—C26	40.5 (4)
C3—C4—C5—C10	−1.2 (4)	C15—C16—C25—C30	−136.8 (3)
C4—C5—C6—C7	−177.9 (3)	C16—C17—C18—C13	−1.9 (4)
C4—C5—C10—C1	−0.3 (4)	C16—C17—C18—C19	−179.1 (2)
C4—C5—C10—C9	177.6 (2)	C16—C25—C26—C27	−176.2 (3)
C5—C6—C7—C8	0.1 (4)	C16—C25—C30—C29	175.0 (3)
C6—C5—C10—C1	−179.7 (2)	C17—C16—C25—C26	−141.7 (3)
C6—C5—C10—C9	−1.9 (4)	C17—C16—C25—C30	41.0 (4)
C6—C7—C8—C9	−1.4 (4)	C17—C18—C19—C20	−138.7 (2)
C7—C8—C9—C10	1.1 (4)	C17—C18—C19—C24	41.2 (3)
C7—C8—C9—C11	178.4 (3)	C18—C13—C14—Cl1	179.04 (18)
C8—C9—C10—C1	178.6 (2)	C18—C13—C14—C15	0.3 (4)
C8—C9—C10—C5	0.6 (4)	C18—C19—C20—C21	−178.3 (2)
C8—C9—C11—O1	0.2 (5)	C18—C19—C24—C23	177.4 (2)
C8—C9—C11—C12	−179.5 (3)	C19—C20—C21—C22	0.4 (4)
C9—C11—C12—N2	−177.5 (2)	C20—C19—C24—C23	−2.7 (3)
C9—C11—C12—C1	2.4 (2)	C20—C21—C22—C23	−1.7 (4)
C10—C1—C2—C3	0.3 (4)	C21—C22—C23—C24	0.8 (4)
C10—C1—C12—N2	178.0 (3)	C22—C23—C24—C19	1.4 (4)
C10—C1—C12—C11	−2.0 (2)	C24—C19—C20—C21	1.8 (3)
C10—C5—C6—C7	1.5 (4)	C25—C16—C17—C18	−176.4 (2)
C10—C9—C11—O1	177.7 (3)	C25—C26—C27—C28	0.7 (4)
C10—C9—C11—C12	−1.9 (3)	C26—C25—C30—C29	−2.3 (4)
C11—C9—C10—C1	0.7 (3)	C26—C27—C28—C29	−1.5 (5)
C11—C9—C10—C5	−177.3 (2)	C27—C28—C29—C30	0.4 (5)
C12—N2—C13—C14	−72.0 (3)	C28—C29—C30—C25	1.6 (5)
C12—N2—C13—C18	112.9 (3)	C30—C25—C26—C27	1.2 (4)

### Hydrogen-bond geometry (Å, º)

Cg1 and Cg2 are the centroids of the C25–C30 and C19–C24 rings, respectively.

**Table:**

*D*—H···*A*	*D*—H	H···*A*	*D*···*A*	*D*—H···*A*
C20—H20···N2	0.93	2.50	2.909 (3)	107
C24—H24···N2^i^	0.93	2.59	3.338 (3)	138
C4—H4···*Cg*1^ii^	0.93	2.74	3.551 (3)	147
C6—H6···*Cg*2^iii^	0.93	2.92	3.647 (3)	136

Symmetry codes: (i) −*x*+1/2, *y*+1/2, −*z*+1/2; (ii) *x*−1/2, −*y*+1/2, *z*−1/2; (iii) −*x*+1, −*y*+1, −*z*+1.
